# Effect of lock boxes and education on safe storage of medications

**DOI:** 10.1186/s40621-020-00257-y

**Published:** 2020-06-12

**Authors:** Alicia C. Webb, Michele H. Nichols, Nipam Shah, Kathy W. Monroe

**Affiliations:** 1 Division of Pediatric Emergency Medicine, Department of Pediatrics, University of Alabama-Birmingham, Birmingham, AL 35233 USA; 2 Children’s of Alabama Hospital, Birmingham, USA

**Keywords:** Medication, Storage, Safe, Lock box, Opioids

## Abstract

**Background:**

Safe medication storage is a proven barrier to ingestions in the pediatric population, but caregivers are often unaware of the importance of safe storage practices or do not have a safe place to store medications. Caregivers may also not be fully aware that the patterns of opioid use and misuse have recently reached crisis levels. The objectives of this study were to define medication storage practices and knowledge of the opioid crisis of participants and to assess the effectiveness of an emergency department intervention on safe medication storage.

**Methods:**

This was a prospective interventional study of caregivers in an urban pediatric emergency department (ED) on safe medication storage and the opioid crisis. Questions assessed the caregivers’ current perceptions and practices related to medication storage and disposal, and opioid crisis awareness. The intervention included verbal instruction about recommended safe storage methods and the opioid crisis, provision of a medication safety handout, and distribution of a medication lock box. A follow-up phone survey conducted 2 weeks later asked participants about use of and satisfaction with the lock box. Frequencies of safe storage behaviors were calculated, and the chi-square statistic was used to compare storage behavior after the intervention.

**Results:**

Fifty caregivers of 112 children were enrolled. Only 4% reported they currently stored medications in a locked or latched place. Thirty-eight percent reported their main barrier to storing medications safely was that they did not have a locked or latched storage location. Fifty percent were unaware of the opioid crisis. Ninety-two percent reported they would use a lock box if given one. Twenty-eight participants (56%) responded to the follow-up phone call survey 2 weeks later. At follow up 90% (25/28) reported they placed their medications within the provided lock box (*p* < 0.00001). Ninety-two percent reported being “very satisfied” with the lock box and how it works.

**Conclusions:**

Despite widespread reporting on this issue, many caregivers remain unaware of safe medication storage practices and the opioid crisis. Providing medication lock boxes removes a commonly reported barrier to safely storing medications and improved reported practices.

## Background

According to recently published Centers for Disease Control and Prevention (CDC) data, unintentional poisoning is among the top 10 causes of both nonfatal and fatal injury for younger children 0–4 years old and is among the leading causes of fatal injury for older children 10–24 years old (CDC 2019a). Patients 0–19 years old comprised 59% of the 2.1 million calls received by United States (U.S.) Poison Control Centers in 2017, and 77% of all calls that year were for unintentional ingestions (2017 Annual Report of the American Association of Poison Control Centers’ National Poison Data System (NPDS) 2018). Exposure to analgesics accounted for the greatest number of reports, though other common household prescription and non-prescription medications were also implicated in large numbers of exposures. Given that unintentional or exploratory ingestions in large part should be preventable, there is clearly room for improvement in current ingestion prevention techniques. Ultimately decreasing children’s access to potentially harmful medications with safe medication storage, including with the use of a medication lock box, can potentially decrease their risk of intentional or unintentional poisoning.

Previous studies have suggested medications are often stored in an unsafe manner. One 2017 study using a web-based survey tool suggested approximately one third of caregivers with children less than 6 years old and only 11% of caregivers with children 7–17 years old reported safe storage practices (McDonald et al. 2017). A 2013 study examining national data from Poison Control Centers and comparing it to the National Center for Health Statistics National Hospital Ambulatory Medical Care Survey (NHAMCS) showed a strong association between national prescribing patterns of opioids with poisonings in children (Burghardt et al. 2013). Additionally, a 2017 study utilized Canadian state databases and reporting systems to show an increased risk of opioid toxicity in children less than 10 years old whose mothers were prescribed opioids (Finkelstein et al. 2017).

The objectives of this study were to evaluate baseline knowledge and behaviors and to determine the impact of a brief emergency department (ED) educational intervention with medication lockbox provision on medication storage practices. Given the prevalence of prescription opioids nationwide and the high levels of toxicity associated with ingestion of these medications, a secondary aim was to determine how many households contained opioid medications and how concerned families were about the opioid crisis. Our hypothesis was providing a medication lock box, thereby removing a commonly-reported barrier to safe medication storage, would improve safe storage practices.

## Methods

### Study design and setting

A pre- and post-interventional study was conducted in the ED of an urban free-standing pediatric hospital in Alabama. Caregivers of patients in the ED were approached by one of the investigators after their child had been triaged as having a non-urgent medical complaint and were placed in a private ED room. A brief explanation of the study and its purposes was given. Verbal consent was obtained from the caregivers to participate in the study. To ensure informed participation, families were excluded if their primary language was any language other than English or if their child was triaged into a critically ill category. First, we administered a pre-intervention 20-question survey about their medication storage practices and beliefs, with an additional focus on opioid medications. The investigator read each question aloud and participants’ answers were recorded using Survey Monkey®, an online database. Information regarding the caregiver’s age, race, level of education, relationship to the patient, current method of storage of medications, and perceptions regarding the likelihood of ingestion of stored medications by pediatric patients in the home was collected. Additional questions were included regarding the participant’s awareness of and concern about the opioid crisis. The study was approved by the Institutional Review Board and relied on verbal consent. Funding was provided by the institution’s Division of Pediatric Emergency Medicine within the Department of Pediatrics.

### Intervention

After the pre-intervention survey, the investigator presented a brief verbal educational piece designed by the research team about the role of safe storage practices in preventing pediatric ingestions in children of all ages, which also included information regarding the opioid epidemic. The participants were also given printed information as a handout with safety tips regarding medication practices pertinent to homes with children published by the American Academy of Pediatrics (AAP). Participants were also given a list of local sites participating in medication take-back programs where caregivers would be able to dispose of leftover medications. At the end of the survey, a medication lock box was provided to participants free-of-charge with instructions on how to use this lock box to secure all home medications. We distributed medication lockboxes using a numerical combination lock to prevent loss of a physical key, as this had been anticipated as a possible barrier to using the lockbox.

### Post-intervention survey

Two weeks following enrollment and the educational intervention, the investigator contacted participants for a post-intervention survey. The questions included: 1) whether the caregiver had received a lock box at time of entry into the study; 2) whether the lock box was used for home medications; and 3) if the lock box was used, the level of their satisfaction with the lock box. Caregivers answering “no” to using the lock box were asked for reasons why it wasn’t being used. Frequencies of study participant characteristics and responses to the pre-and post-intervention surveys were calculated. The Chi-square statistic was used to compare safe medication storage before and after the intervention in this caregiver study population.

## Results

Fifty caregivers were surveyed, representing a total of 112 children. The racial composition of the study sample (Table 1) was similar to the overall ED patient population (41% Caucasian, 51% African American). Eighty percent of survey participants were mothers, and 92% lived with the patient all of the time. One-third of caregivers were the only adult within the home. The distribution of ages of the children in the home was 37% 0–5 years, 38% 6–11 years, 21% 12–17 years, and the remaining 4% were 18 and older.

**Table 1:** Demographic data reported by participating caregivers

Demographic	***n*** = 50	%
White or Caucasian	17	34%
Black or African American	32	64%
Other	1	2%

**Fig. 1:**
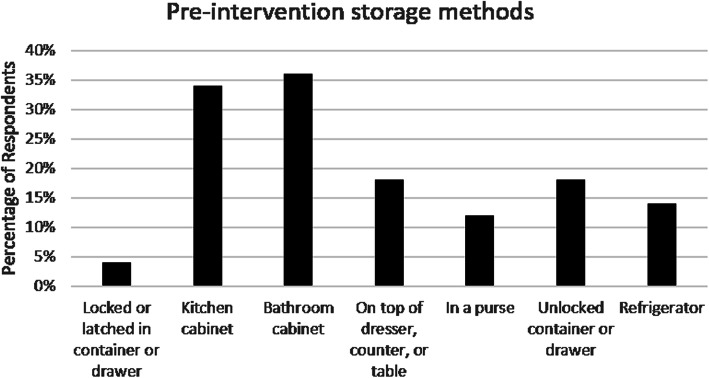
Pre-intervention methods of medication storage

Only 4% of caregivers (2/50) reported currently storing medications in a locked or latched container or cabinet. Figure 1 shows the common storage areas reported, with kitchen or bathroom cabinets being the leading sites. Despite such low rates of current safe medication storage practices, 56% (27/50) believed it was either unlikely or very unlikely their child or their child’s friends could gain access to prescription medications in the home. Almost 40% (18/48) of caregivers reported their main barrier to storing medications safely was they did not have a locked or latched place to store them. Twenty-six percent (13/50) reported they had never thought about where they stored their medications. Ninety-two percent of caregivers reported they would use a medication lock box if given one.

**Fig. 2:**
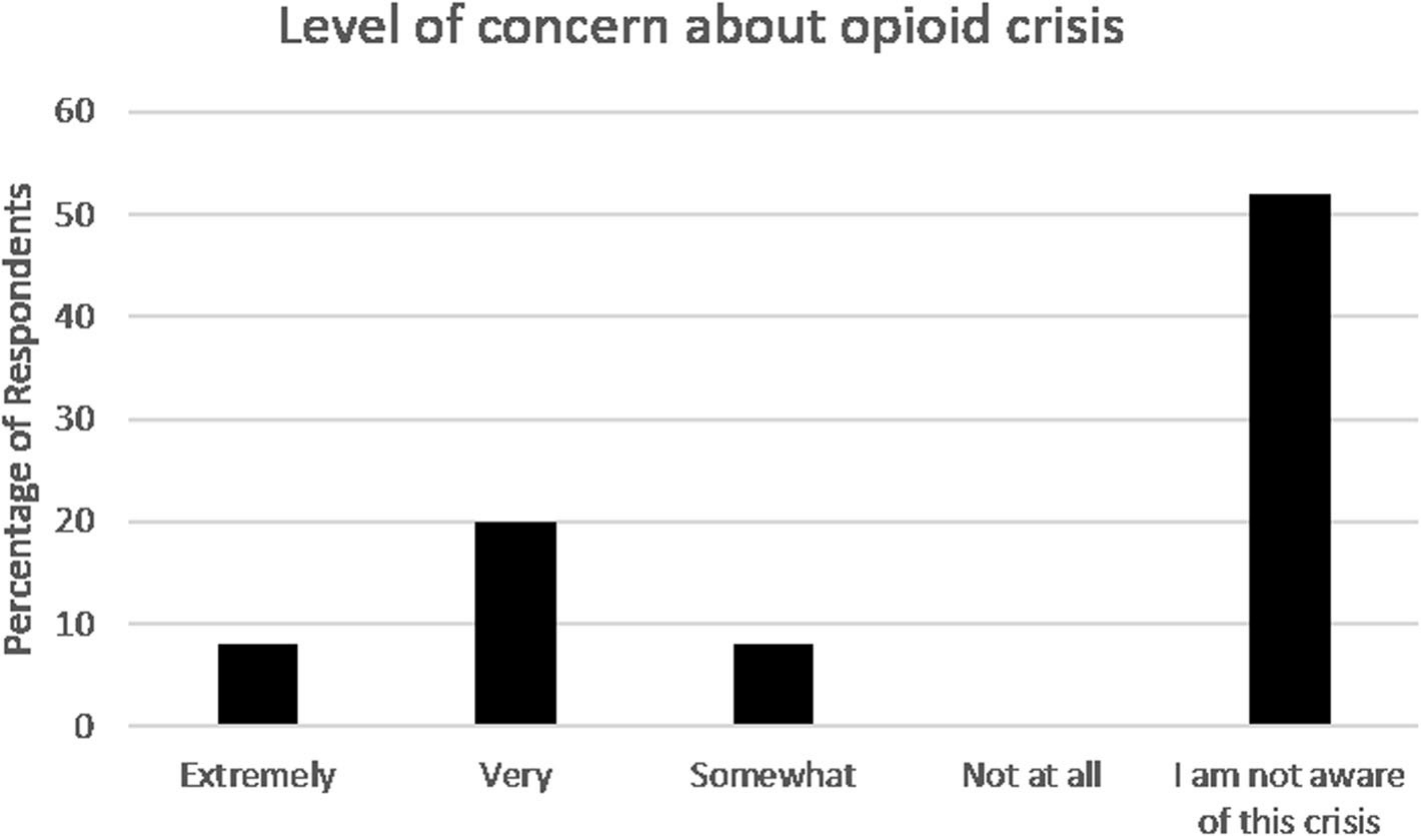
Level of concern about the opioid crisis

Prescription opioid medications had been in the home of 38% (19/50) of respondents within the last 12 months, and of those, 52% reported no one in the home was currently using these medications. Only 16% of caregivers reported a safe method of medication disposal when asked how they would get rid of expired prescription opioids if needed. When asked about their level of concern regarding the opioid crisis, 8% reported they were extremely concerned, 12% reported they were very concerned, and 52% reported they were not aware of this crisis (Fig. 2).

At follow up 2 weeks later, 28 participants (56%) were successfully reached via telephone. All reported receiving a medication lock box during the ED intervention, which confirmed we were speaking with the correct individual and they recalled our encounter. Ninety percent of participants (25/28) reported they had placed their medications within the provided lock box. The increase in reported safe storage methods was statistically significant (*p*-value < 0.00001). Of those who placed their medications within the lock box, 92% reported they were very satisfied, and 8% reported they were somewhat satisfied with the lock box and how it worked.

Of those who were not using the lock box for safe medication storage, one reported she had been out of town since the encounter and was planning on placing medications within her lock box on arriving home. The other participant not using the lock box reported she and her family were instead using the lock box to store the keys to their gun safe as an added layer of protection against unintentional firearm injury. Multiple caregivers reported they had used the encounter as an opportunity to go through the home and properly dispose of unused medications. One participant also reported she had watched her young daughter pick up the lock box and unsuccessfully try to open it, which reinforced to her the benefit of having a locked place to store medications.

## Discussion

In our study focused on the safe storage of medications, we found an extreme minority (only 4%) of participants initially reported storing medications in a locked or latched place, and yet most caregivers felt that they were doing enough to prevent their children from accessing their medications. After our educational intervention and medication lock box distribution, safe medication storage practices increased in our caregiver study population. Because actual rates of safe medication storage may in practice be even lower than those self-reported in our surveys, this speaks to the frequency of a dangerous home environment placing children at risk of exposure to these medications. There was a high degree of satisfaction with the provided lock box at the time of follow-up.

Our study demonstrated a brief intervention consisting of verbal and written education and a free safety device as part of a visit to an emergency department was successful, with significantly improved reported safety practices at follow up. Educating parents on methods of keeping their children safe is the first step in injury prevention. Previous studies aimed at assessing the effectiveness of various injury prevention strategies have had mixed results. A study published in 2002 comparing an educational intervention paired with access to reduced-cost safety equipment to in-home visits showed no additional benefit of a home visit (Gielen et al. 2002). However, they were able to show a statistically significant improvement in observed safety practices in those who had made use of a community safety center designed to improve access to affordable safety equipment compared to those who had not utilized this resource. Another study used a randomized controlled trial of home safety interventions in which members of the research team conducted home surveys and installed safety devices within the homes of participants, which demonstrated a 70% reduction in injury among the study group (Phelan et al. 2011). While home observation would yield the most accurate data regarding medication storage practices, the resources required makes this approach challenging. Additionally, even without a home visit component, several studies have concluded the combination of education plus the provision of free or low-cost safety equipment is an effective way to promote injury prevention practices (Achana et al. 2015; Kendrick et al. 2012).

A secondary finding of our study showed a large portion of households contained prescription opioids within the preceding 12 months. The opioid crisis has been identified as a public health emergency, (Report of the President’s Commission on combating drug addiction and the opioid crisis 2017) yet only half of the participants in our study were aware of the epidemic. This is similar to findings reported in which 53% of the public considers addiction to prescription pain medication a major problem, but only 28% would consider it a national emergency (Blendon and Benson 2018). According to the Centers for Disease Control and Prevention (CDC), prescription opioids were involved in 47,600 deaths nationally in 2017 and accounted for 68% of all overdose deaths (CDC 2019b). Death rates related to prescription opioids were five-fold higher in 2017 compared to 1999. Additionally, access to prescription opioids has skyrocketed over the last two decades. In 2017 there were 58 opioid prescriptions written for every 100 Americans, totaling 191 million prescriptions per year. And while there is clearly a nation-wide problem, this crisis disproportionately affects certain areas of the country. Prescribing practices from 2017 broken down by state shows Alabama at the top, with 107 opioid prescriptions per 100 persons in 2017 (CDC 2019c). It follows that if there are more opioids in the state than anywhere else in the country, Alabama children are at high risk of exposure to these potentially dangerous medications through intentional or unintentional means. This underscores the importance of not only relying on nationally based programs, but also implementing targeted injury prevention interventions specific to local and state-wide challenges.

### Limitations

A major limitation of this study design is the actual medication storage behavior was not observed, but relied on self-reporting of behaviors through in-person baseline and phone follow up interviews. Even with this inherent bias that might inflate reported rates of safe practices, the pre-intervention storage behaviors found in our patient population were still extremely unsafe. Another limitation of the study design was participation in the follow-up survey relied on caregivers answering the follow-up phone calls with about half of the original study population completing follow up. In theory, the attrition rate could have resulted in some of the less motivated participants not being captured due to their lack of answering the phone. Additionally, only English-speaking caregivers were approached for participation in the survey, which does limit generalizability to U.S. populations that are not proficient in English. The physical location of the study was in one urban pediatric hospital with a limited sample size, especially for follow up. Nonetheless, we are able to report a statistically significant increase in safe medication storage practices. Due to the unique geography of the location, the patient population comes from urban, suburban, and rural communities throughout the state and as such may less influence the limited generalizability of the results.

## Conclusions

Few families store medications safely, and based on the results of our study, it seems to be from a lack of knowledge about what practices are safe rather than from a lack of concern. Despite widespread reporting of the opioid crisis in medical and layperson arenas, many caregivers remain unaware of the importance of safely storing medications and of the opioid crisis. An ED based medication safe storage intervention including education and provision of a free lockbox dramatically improved reported medication storage practices. In a state where access to opioids is so widespread, it is especially important for the people of Alabama to participate in targeted interventions aimed at improving medication storage practices.

## Data Availability

We are happy to make our data and surveys available upon request.

## Abbreviations

AAP: American Academy of Pediatrics
CDC: Centers for Disease Control and Prevention
ED: Emergency Department
NHAMCS: National Hospital Ambulatory Medical Care Survey
U.S.: United States
